# Analyzing the Implementation of Policies and Guidelines for the Prevention and Management of Type 2 Diabetes at Primary Health Care Level in Nepal

**DOI:** 10.3389/fpubh.2022.763784

**Published:** 2022-02-09

**Authors:** Rabina Shrestha, Uday Narayan Yadav, Abha Shrestha, Grish Paudel, Deepa Makaju, Prakash Poudel, Hanako Iwashita, Yuriko Harada, Archana Shrestha, Biraj Karmacharya, Rajendra Koju, Tomohiko Sugishita, Lal Rawal

**Affiliations:** ^1^Department of Community Programs, Dhulikhel Hospital Kathmandu University Hospital, Kathmandu University, Dhulikhel, Nepal; ^2^National Centre for Epidemiology and Population Health, Research School of Population Health, The Australian National University, Canberra, ACT, Australia; ^3^Centre for Primary Health Care and Equity, University of New South Wales, Sydney, NSW, Australia; ^4^School of Health, Medical and Applied Sciences, College of Science and Sustainability, Central Queensland University, Rockhampton, QLD, Australia; ^5^Centre for Oral Health Outcomes & Research Translation (COHORT), School of Nursing and Midwifery, Western Sydney University, Sydney, NSW, Australia; ^6^Department of International Affairs and Tropical Medicine, Tokyo Women's Medical University, Tokyo, Japan; ^7^Physical Activity Research Group, Appleton Institute, Central Queensland University, Rockhampton, QLD, Australia; ^8^Translational Health Research Institute, Western Sydney University, Sydney, NSW, Australia

**Keywords:** guidelines, implementation, Nepal, policy, type 2 diabetes, prevention, management

## Abstract

**Background:**

Nepal, in recent years, is witnessing an increasing problem of type 2 diabetes that has resulted significant premature deaths and disability. Prevention and management of non-communicable diseases (NCDs) including diabetes have been prioritized in the national policies and guidelines of the Nepal Government. However, research looking at the overview of the implementation of the existing policies and guidelines for diabetes prevention and control is scarce. Hence, this study reviewed diabetes related existing policies and its implementation process at the primary health care level in Nepal.

**Methods:**

This study involved two phases: Phase I: situation analyses through review of documents and Phase II: qualitative exploratory study. In phase I, four databases (Medline, Web of Science, Embase and PubMed) were systematically searched using key search terms related to diabetes care and policies between January 2000 and June 2021. Also, relevant gray literature was reviewed to understand the trajectory of policy development and its translation with regards to diabetes prevention and management at primary health care level in Nepal. Following the phase I, we conducted in-depth interviews (IDI) and key informant interviews (KII) with health care providers, policy makers, and managers (IDI = 13, and KII = 7) at peripheral and central levels in Kavrepalanchowk and Nuwakot districts of Nepal. The in-depth interviews were audio recorded, transcribed, and coded. The triangulation of data from document review and interviews was done and presented in themes.

**Results:**

Four key themes were identified through triangulating findings from the document review and interviews including (i) limited implementation of policies into practices; (ii) lack of coordination among the different levels of service providers; (iii) lack of trained human resources for health and inadequate quality services at the primary health care level, and (iv) inadequate access and utilization of diabetes care services at primary health care level. Specifically, this study identified some key pertinent challenges to the implementation of policies and programs including inadequate resources, limited engagement of stakeholders in service design and delivery, lack of trained health care providers, lack of financial resources to strengthen peripheral health services, fragmented health governance, and weak reporting and monitoring systems.

**Conclusion:**

This study revealed that the policies, plans, and strategies for prevention and management of NCDs in Nepal recognized the importance of diabetes prevention and control. However, a major gap remains with adequate and lack of clarity in terms of implementation of available policies, plans, strategies, and programs to address the problem of diabetes. We suggest the need for multisectoral approach (engaging both health and non-health sectors) at central as well as peripheral levels to strengthen the policies implementation process, building capacity of health care providers, ensuring adequate financial and non-financial resources, and improving quality of services at primary health care levels.

## Introduction

Diabetes mellitus is one of the most prevalent chronic conditions associated with increasing morbidity, mortality, and economic burden worldwide (1). In 2019, there were 463 million adults living with diabetes worldwide, and this number is estimated to reach 700 million by the year 2045 (2). An additional 374 million people had prediabetes and were at a risk of developing type 2 diabetes (2) The burden of diabetes and its impact on life of the people is substantially higher in low middle income countries (LMICs), in comparison to high-income countries (3). The United Nations (UN) Sustainable Development Goals (SDG-3 Health and Wellbeing); target 4 that focuses on prevention and treatment of non-communicable diseases (NCDs), has prioritized in reducing the global burden of NCDs including diabetes. Likewise, the WHO (World Health Organization) Global Action Plan on NCDs (2013–2020) has emphasized to raise the priority in the national and international agendas and goals to the prevention and control of NCDs, through strengthened international cooperation and advocacy” (4).

South Asia, where a quarter of the world population resides, has a majority of all global diabetes cases (5). Nepal, a South Asian developing country has a high prevalence of both prediabetes (9.2%) and diabetes (8.5%) cases (6). However, the burden of diabetes is likely underreported because the peripheral health system of Nepal has not yet been well equipped with the infrastructure to screen, diagnose, and treat the cases with diabetes (7–10). Despite the polices and guidelines in prevention and management of diabetes in Nepal, there are significant challenges, including limited health care facilities and medications within the current health care system and lack of awareness about diabetes and healthy lifestyle related behaviors among people (8–10).

Being cognizant of the impacts of NCDs including diabetes, the Government of Nepal (GoN), in recent years, has developed some policies, guidelines, and manuals to guide the prevention and management of NCDs including diabetes. The Government of Nepal annual report shows that different level of NCDs related services are being provided at local health facilities, such as services for hypertension and diabetes detection, counseling, referral, follow up services for NCDs patients including diabetes, drugs refilling, and health promotion related services (11). This initiative reflects the greater effort of the Government of Nepal toward the prevention and control of diabetes. The Government of Nepal, in recent years, has launched a multisectoral Action Plan for the prevention and control of NCDs and implemented PEN (package of essential non-communicable disease) in selected districts of Nepal.

Despite the development of policies and strategies on NCDs including diabetes prevention and control in the country, the evidence on the implementation of these policies remained largely unexplored. Therefore, in this study, we systematically reviewed the existing literature that included both academic literature and policies specific to diabetes in Nepal and then conducted a qualitative study, to explore the implementation aspects of policies and strategies related to diabetes prevention and management in Nepal. The study aimed to answer two research questions: (i) What are the policies and guidelines being developed to prevent and control type 2 diabetes in Nepal (ii) what is the implementation status of the existing policies and guidelines for diabetes prevention and control in Nepal?

## Methods

This study adopted an approach of combining documents review and in-depth interviews (IDI) and key informant interviews (KII) to answer the research questions. This approach has been widely adopted in the previous studies to identify the factors in the inclusion and prioritization of NCDs in polices, guidelines, and actions plans (12–15). In phase I, we systematically searched and reviewed the relevant published academic literature and gray literature from websites of Ministry of Health to understand the trajectory of policy development and its translation with regards to diabetes in real world settings in Nepal. In phase II, we conducted in-depth interviews and key informant interviews with health care providers, policy makers, and managers. Details of the phase wise study are as follows.

### Phase I: Situation Analyses Through Literature Review

In this phase, a systematic search was conducted in four key electronic databases including Medline, Web of Science, Embase, and PubMed to identify peer-reviewed journal articles and government documents (plans, policies, and guidelines). Also, gray literature was searched through google and webpage of the Ministry of Health and Population, Government of Nepal. The literature search was limited to the academic and gray literature published between 01 January 2000 and 31 June 2021. Most policies for NCDs prevention and control in Nepal have been formulated in recent decades (11). The National Nutrition Policy and Strategy initiated in 2004 also highlights the healthy eating pattern including household food security, improved dietary habits, and preventing lifestyle related diseases (16). We searched gray literature (particularly NCDs) related policies, and plans published in English as well as Nepali.

We considered search of literatures by using combinations of different keywords, namely “diabetes,” “people with diabetes,” “diabetes mellitus,” “sugar,” “guidelines,” “protocol,” “policy,” “guide,” standard practice,” “strategy,” “implementation,” “intervention,” “Nepal,” “health post,” “primary health care center,” “peripheral health system,” “facilitators,” “Barriers,” and “community health workers.”

#### Inclusion Criteria

The inclusion criteria of this study is:(i) The primary or secondary studies (both quantitative and qualitative) focused on diabetes prevention only, or management or prevention and management in Nepal; (ii) programs that cover health promotion, prevention, treatment, or rehabilitation activities for people with diabetes at the primary health care center or health post or community level (iii) Primary or secondary studies that focused on barriers and facilitators for management of diabetes in Nepal, and (iv) policies and plans documents focusing on NCDs in Nepal.

#### Exclusion Criteria

The exclusion criteria is: (i) studies including Gestational Diabetes Mellitus or Diabetes Insipidus (ii) studies that solely focused on estimating prevalence of diabetes in Nepal, (iii) clinical interventions studies of diabetes prevention or management, and (iv) studies based on hospital settings.

#### Data Extraction and Synthesis

The data extraction and synthesis process as recommended and by Heller et al. (12) and other researchers (13, 14) was adopted. The data obtained from the search and review of documents were extracted and presented in the narrative form. The key components/areas of the documents review were identified according to the objectives of the study. These include implementation of policies and strategies into practice, coordination among the different level of health care providers, primary health care challenges in delivering comprehensive care, and boundaries for the people with diabetes to access diabetes care at primary health care.

The results of document review guided the development of interview guide and inform the identification of the potential participants needed for the study to explore the implementation process of the existing policies and guidelines for diabetes management from the perspective of health care workers working with peripheral health system of Nepal.

### Phase II: Exploratory Study

#### Study Setting

The qualitative study was conducted in Kavrepalanchowk and Nuwakot districts of Nepal. Kavre is one of the 77 districts of Nepal with Dhulikhel as its district headquarters and covers an area of 1,396 km^2^ (539 sq mi). Kavrepalanchok with an area of 1,396 sq.ft. is situated at a mid-hilly area majorly, having the subtropical climate with an elevation range of 280–3,018 m, bordered to the east by Ramechhap and Dolakha, west by Kathmandu valley, north by Sindhupalchok, and south by Sindhuli and Makawanpur. According to CBS, 2068, the total number of households is 80,720 with a total population of 381,937 including the number of men 182,936 (47.90%) and women 199,001 (52.10%). The adult literacy percentage is 62.77% and per capita income is $1399 (17). The people in this district rely upon income from the business and agricultural farming (18).

Nuwakot district is located in the Bagmati zone, covering an area of 1,121 km^2^ in the mid development region of Nepal. According to the national census 2011, the total population of the district is 2,77,471 comprising 1,32,787 men and 1,44,684 women. Of these, 54.6 spoke Nepali, 39.9 Tamang, 2.0 Newari, and 0.9% Lepcha as their first language (19). Agriculture, service, and industry are the major activities performed by the people of this district. A little over half of the (51.25%) population in this district is engaged in agriculture and 14.43% in some form of employment (20).

In this phase, IDIs (*n* = 13) and KIIs (*n* = 7) were conducted in two districts namely Kavrepalanchowk (*n* = 13) and Nuwakot (*n* = 7). These two districts were chosen due to the convenience for the researchers to access, where the study team has been implementing diabetes management intervention using the Randomized Controlled Trial. The interviews included 13 IDIs with health care providers at community level and 7 KIIs with health policy makers and program managers at central and district levels. The general characteristics of the participants involved in in-depth and key informant interviews are presented in Table 1.

**Table 1:** General characteristics of the In depth interview/Key infromant interviews participants.

**Characteristics**	**Total**
In-depth interviews	13
Key Informant Interviews	7
**Work setting**	
Rural municipality/ Rural health centers	8
Urban municipality/ Urban health centers	9
Central/ policy level representative	1
District health facility	2
**Age by category**	
20–29	1
30–39	9
40–49	6
≥50	4
**Gender**	
Male	15
Female	5
**Duration of job in the public health facilities**	
<10 years	10
10–19 years	3
20–29 years	7

We adopted purposive sampling to recruit the participants. A semistructured interview guide was used to obtain the data. The focus areas included in the interview schedule were (i) access, availability, and utilization of services for prevention and management of diabetes; (ii) facilitators and barriers to the prevention and management of diabetes; (iii) potentials to developing culturally appropriate and context specific intervention approaches to prevention and management of diabetes. Telephone interviews were conducted due to the COVID-19 restrictions. All the interviews were conducted in the Nepali language by two members of the research team (RS and DM). All in depth interviews and key informant interviews were audio recorded and were transcribed verbatim. The transcripts were then translated into English by a research team member (RS) and checked for accuracy by another team member (UNY). Three interview transcripts were randomly selected and checked by additional team members (UNY and LR) to ensure reliability of the transcription.

#### Data Processing and Analysis

The transcripts were individually read and re-read several times in order to become familiar with the data and to record initial ideas then organized into a coding framework, informed by the interview topic guide. A thematic analysis using an inductive approach was used to identify the emerging patterns and themes within the data (2017) (21). Themes were identified by comparing and contrasting the patterns and meanings as expressed by the participants. Two researchers (RS and DM) independently coded the transcripts and identified the themes which were further checked by another two researchers (UNY and AS) for accuracy. All project team members reviewed the final themes, and a consensus was achieved resolving the discrepancies through discussion. A final set of three major themes was identified and the qualitative findings are presented, accordingly. These include (i) access to and utilization of NCDs including the diabetes related programs in Nepal, (ii) challenges and enablers for prevention and management of diabetes in Nepal, and (iii) potentials to developing culturally appropriate and context specific intervention approaches to prevention and control of diabetes in Nepal. The triangulation of data from document review and interviews was guided by the data extraction and synthesis process as recommended by Heller et.al (12) and other researchers (13, 14).

The reporting of this qualitative research adhered to the Standards for Reporting Qualitative Research (SRQR) (22). Several strategies were employed to enhance the trustworthiness (credibility, transferability, dependability, confirmability, and transferability) of the study findings (23, 24). This included checking the data for accuracy, organizing debriefings for completeness of data (RS, DM, UNY and, LR). See Supplementary Material, SRQR Checklist.

#### Ethics Approval

This study is a part of an ongoing larger research project in Nepal, which uses a randomized controlled trial for diabetes self-management in Nepal. Ethics approval of the main study has been obtained from the Ethics Review Committees of Nepal Health Research Council, Nepal, Tokyo Women's Medical University and Central Queensland University Australia. Informed verbal consent was obtained from all participants prior to audio recordings of the interview.

## Results

**Figure 1:**
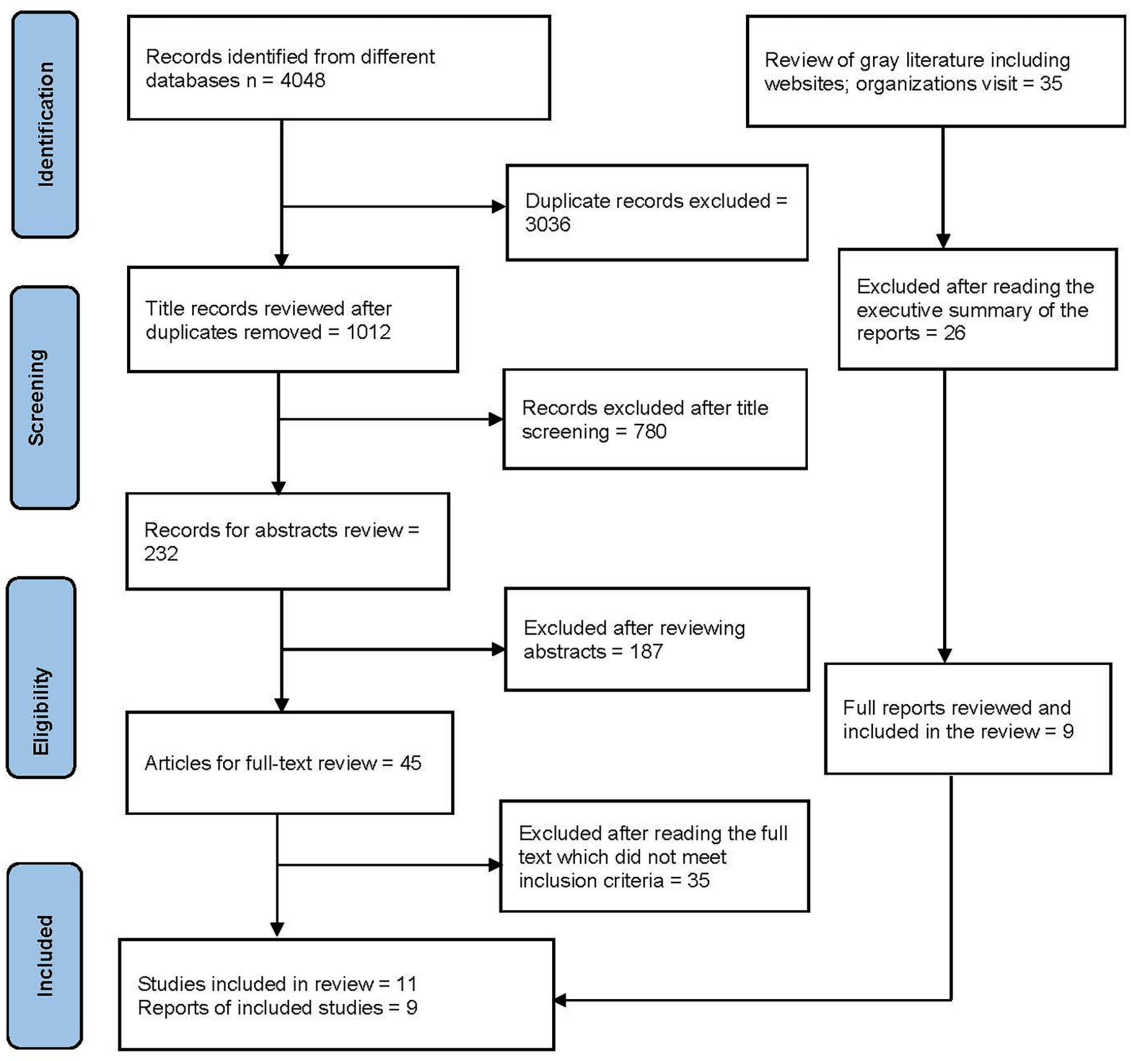
Systematic search and review flow diagram using PRISMA.

The systematic search of different databases yielded 4,048 studies. A total of 1,012 (not duplicated) publications were screened. The titles and abstracts were screened for 232 studies and after removing the publications/ reports that did not meet inclusion criteria, 45 studies met full-text review criteria. The full text records/documents were reviewed and further assessed for eligibility and finally, 11 records met the inclusion criteria and were included in this review. In addition, a total of 9 records were retrieved from the gray literature search and review of citations/reference lists. Altogether, 20 relevant studies and policy documents were included in the review (see Figure 1). We have also summarized the findings obtained from the documents review in the tables (see Tables 2, 3), the data obtained from documents review and exploratory research were triangulated, and the findings are presented in key thematic forms.

**Table 2:** Policies and strategies relevant to diabetes prevention and control in Nepal.

**SN**	**Organization/ year**	**Name of policies/plan/Act**	**Diabetes related information and guiding principles**
1	Ministry of Health and Population, Government of Nepal, 2019	National Health Policy, Nepal 2019	• The individuals, family, community and related institutions will be made responsible for prevention and management of NCDs. Policy have highlighted the implementation of healthy lifestyle promotion at all levels of the health care facilities. • Ensuring the management such as the treatment of NCDs will be available from primary level to referral level hospitals. • Inclusion of Multisectoral Action plan and PEN package in the strategy of NCD.
2	Ministry of Health and Population, Government of Nepal, 2014	Multi-sectoral Action Plan for Prevention and control of noncommunicable Diseases (2014–2020)	• Prioritizing the prevention and control of non-communicable diseases in all policies. • Accelerating country response for the prevention and control of NCDs through strengthening national capacity, leadership, governance, multisectoral action and partnership. • Monitoring the trends and determinants of NCDs and evaluating their prevention and control program.
3	Department of Health Services, MoHP, Nepal, 2017	Package of Essential Non-Communicable Disease Intervention at Primary Health Care Setting	• Protocols for behavioral interventions to address key modifiable risk factors: tobacco cessation, dietary modification, avoiding harmful use of alcohol and increasing physical activity, which can be delivered through an integrated approach.
4	Ministry of Health and Population, Government of Nepal, 2016	Nepal Health Sector Strategy Implementation Plan (2016–2021),	• Target of reducing NCD, the figure of 8, 31,695 to 6, 78, 953 by 2020. • Strengthening health system and governance, improving quality of care at point-of-delivery, decentralized planning and budgeting, promoting healthy lifestyles and increased used of evidence in decision making at all levels.
5	Ministry of Health and Population, Government of Nepal, 2011	Nepal Tobacco Products Control and Regulatory Act, 2011	• At least 25% of the total amount raised from the revenue levied from the excise tax upon smoking and tobacco products by the Government of Nepal as per the financial act shall be deposited in the health fund. • Manufacturer should print the warning message 'Tobacco Product is Injurious to Health' clearly in Nepali and also graphic color pictures showing health hazards of consuming tobacco products on boxes, wrappers, packets, packaging materials and labels covering at least 90% of the space. • Graphic color pictures and warning messages should be printed on the inside as well as the outside of boxes, packets, wrappers, parcels, cartons and packaging materials of cigarette, bidi, chewing tobacco, tobacco and gutkha etc. • Smoking or consuming tobacco in public places, and even in house or private vehicle is not allowed. • Public Notice on smoking prohibition to be displayed. • No person including the manufacturer shall be allowed to advertise and promote or release or sponsor, or disseminate any program, news or information about tobacco products through newspaper and electronic media. • No tobacco selling or gift to a person below age of 18 and to pregnant woman.
6	Nepal Nutrition and Food Security Portal, 2018	The Right to Food and Food Sovereignty Act. 2018	• The Act ensures access to food to the citizens is as fundamental human right. • The Act states implementation of fundamental rights relating to food, food security and food sovereignty of the citizens. • The Act monitors the quality of food so that the people consume healthy foods. • Prohibition on production, sale or distribution of adulterated foodstuff or sub-standard foodstuff • The designated authority may, if he/she suspects that any foodstuff is * adulterated or substandard, withhold such foodstuff upon sealing it and shall receive receipt upon granting custody of such foodstuff to the owner.
7	Government of Nepal, 2007	Food (Fifth Amendment) Rules, 2064 (2007) 2064.6.14 (1 Oct. 2007) (Packaging and labeling, Food Adulteration Act)	• Mandatory labeling on container of packed food along with product description in details and date of manufacture and expiry. • Label not required on the following packed food like Fruits or green leaves kept fresh through the use of gas or cold storage or freezing or any other provision, • Label required on food canned, bottled or subject to other processing system, except in cases where it is kept fresh as mentioned above like Milk in liquid state other than condensed milk, Whole egg, Various kinds of fishes, meat other than the fish or food containing meat sealed in a can or bottle, Provided that, in the case of meat, it shall clearly indicate in writing the meat of which animal. • No person shall sell the flesh of any animal having died a natural death or a food containing such flesh or keep such flesh or food for sale.
8	Ministry of Health and Population, Nepal, 2017	Alcohol Regulation and Control Policy, 2017, Multisectoral Action Plan	• The core features of the new law are: • Total ban of alcohol advertisement, promotion and sponsorship. • Decreasing availability: in the future alcohol will only be sold by especially licensed shops for certain hours. • Decreasing alcohol availability: the minimum age for alcohol purchases is increased from 18 to 21 years. • All alcohol containers will have at least a 75% health warning. Nepal will be first country in the world to introduce 75% pictorial warning. • Alcohol will no longer be used in Government-sponsored programs and events. • Alcohol is no longer allowed to be sold in public places including heritage sites, educational institutions, and sports complexes.
9	Nepal Law Commission, 2018	The Public Health Service Act, 2018	• Health system strengthening at local, provincial, and federal level. • The Government of Nepal shall make arrangement for providing every citizen with quality health service from a health institution. • The Government of Nepal shall prepare and enforce the policy and standards relating to development, distribution and use of human resources in the health sector. • While prescribing standards referred to in sub-section (1), such standards shall be prescribed on the basis of estimation of human resources, technology and equipment by analyzing and mapping of long-term, mid-term and immediate requirements for the development, management and use of human resource. • The Government of Nepal and the designated government body may conduct regulation, inspection, monitoring, investigation and evaluation as to whether or not the health institution and service provider have maintained the quality pursuant to the prescribed standards.

**Table 3:** Publications related to prevention and control of diabetes in Nepal.

**SN**	**Author/year**	**Study/ document type**	**Objective**	**Key findings**
1	Ide et al. (9)	Qualitative study	To assess the current state of diabetes services provided by health facilities and to identify the major barriers that people with diabetes commonly face in Nepal.	• Lack of patients' knowledge and self-management behaviors. • Scarcity of financial and human resources for health. • Community or cultural barriers, such as the recent shift to diets high in carbohydrates and sugar. • Major gaps remain in the provision of services for diabetes. • Financial barriers continue to prevent some patients from receiving regular treatment, including checkups and medication especially for patients requiring insulin. • Failure in adapting guidelines to the Nepali context.
2	Upreti et al. (26)	Perspective	Assess the strengthening policy and governance to address the growing burden of diabetes in Nepal	• High out-of-pocket expenditure for health. • Need to adopt a public health approach that balances individual and population-level interventions to halt the rise in diabetes and obesity by the government of Nepal. • Need awareness, early diagnosis and prevention for management and control of diabetes. • Lack of nationwide robust programs for diabetes prevention in the country and services are inaccessible to much of the Nepalese population.
3	Khanal et al. (48)		To estimate the cost of quality pharmacological treatment of diabetes in 4Nepal	• The planned health insurance seems unaffordable if patients are treated with the same medicines as those in one of the best healthcare systems in the world. • The government needs to stimulate rational prescribing and secure additional funding through taxation, health insurance or health levy to provide such medicines and services.
4	Ghimire et al. (25)		To assess the readiness of health facilities for cardiovascular diseases (CVDs), diabetes and chronic respiratory diseases (CRDs) services in Nepal.	• Disproportionate lack of human resources, medicines, equipment, and supply chain logistics in remote regions of Nepal. • Patient's socio-economic status, distance to health centers, transportation, direct, and indirect costs associated with attending health centers further compound the utilization of health services in rural regions. • Sub-optimal readiness of services related to three NCDs at the public facilities in Nepal. • Compared to public facilities, private facilities showed higher readiness scores for CVDs, diabetes and CRDs. • Urgent improvement in health services, particularly in public facilities, are critical to manage common NCDs to cope up with the growing burden of NCDs.
5	Kattel et al. (27)	Editorial	To identify the challenges of being a diabetic in Nepal	• Challenge of comprehensive quality care at low cost to patients. • Less or inaccessible the point of care facilities for diabetic patients in Nepal are either compared to the burden of disease. • Increase in the indirect cost of illness of a diabetic patient in terms of travel and accommodation which is again not easier as compared to high income countries. • Inadequate manpower in terms of primary physicians trained on diabetes at primary health care and districts hospitals, and diabetic team (diabetic physician, dietician, counselor and nurses) at zonal and tertiary hospitals. • Lack of laboratories facilities that can monitor beyond plasma sugar level in primary care.
6	Gyawali et al. (10)	Literature review	To review evidence on the prevalence, cost and treatment of diabetes mellitus type 2 and its complications in Nepal and to critically assess the challenges to be addressed to contain the epidemic and its negative economic impact.	• Limited health care facilities. • High cost of treatment: out-of-pocket expenses associated with diabetes. • Lack of disease awareness among patients, • Lack of specific guidelines for the prevention and treatment of diabetes. • Higher priority on communicable diseases and maternal and child health services and by a private health system focused on curative medicine than diabetes treatment and prevention efforts. • An urgent need to strengthen the health system so as to enable it to effectively face the challenges posed by diabetes and other NCDs. • Preventive strategies must take into account the growing prevalence of risk factors associated with these diseases. • An immediate need for a broad-based approach, and involvement of relevant stakeholders from the government, civil society, and the private sector for early detection and primary and secondary prevention of diabetes and its complications. • Need a comprehensive action plan to tackle diabetes and other NCDs with clear responsibilities, a monitoring plan backed by the necessary funds for public awareness about risk reduction behaviors, and availability of essential medicines to all sectors of the community.
7	Baral et al. (50)	Background series-HERD	To identify the current status and challenges and recommend accordingly	• Attempts have been made on revising tobacco taxes and strengthening anti-smuggling measures. • Standardization and mandating the food labeling policy by the government. • Heavy involvement of government in the purchase of essential medications to increase the access and affordability. • Attempts have been made in establishing a regional health technology assessment institution to improve the effectiveness of interventions. • The problem of diabetes is reflected by its increasing complications and lack of access to proper and timely treatment. • In Nepal, traditional dietary patterns are being lost as the population adapts to more industrialized and urban food environments. These changes have a significant impact on type 2 diabetes risks by increasing body weight and central adiposity and decreasing physical activity. • There is historical deficiency in knowledge about diabetes and inequalities in the quality of education reaching each region in the country. • Low level of community knowledge of diabetes reflects on the extent of health promotion for most 4 chronic noncommunicable diseases. • No comprehensive primary care programs for diabetes in the country. • Uncoordinated health promotion efforts by different stakeholders and lack of clear guidelines regarding diabetes education and • Low knowledge of diabetes among health care workers who are expected to deliver health education to the community people.
8	Aryal et al. (36)	Descriptive Cross-sectional study	Assess the status of health facilities to implement the NCDs related programs/ services in Nepal.	• Need for capacity building and enhancement of the health facilities for effective implementation of PEN in Nepal. • The need of human resource to be fulfilled for the delivery of quality services at primary care level. • Need to ensure the availability of all basic equipment at all the levels of health facilities. • At the time of the survey, medicines like Statins, Enalapril and Glibenclamide were not available in any of the health facilities in both districts. This means the emergency situations at health institution may not be managed promptly leading to needless deaths. This demands the need of the strengthening of the health facilities especially at primary health center and health post level to combat NCDs. • Adequate and consistent supply of basic diagnostics, equipment, essential medicines is essential for effective management of NCDs. • Efficient use of the limited health care resources available, availability of basic diagnostics, essential medicines, referral system, and health workforce can equitably deliver the services based on the Primary health care.
9	Sapkota et al. (40)	Qualitative study	To investigate diabetes-related healthcare services provided in Nepal, and explored healthcare professionals' opinions of the barriers to, and strategies for, effective diabetes care	• The shortage of diabetes educators was identified as a major challenge in effective education dissemination and overall diabetes management. • the lack of collaborative efforts between the HCPs- a significant barrier to effective diabetes treatment outcomes. • Multi-professional collaboration efforts in diabetes care in Nepal could not only improve diabetes management, but could help reduce the burden for physicians, decrease patients' dependency on physicians for information and open opportunities for the ‘under-utilized' professions, such as pharmacists and dieticians. • Although a part of the healthcare system, HCPs are significantly affected by the broader healthcare system (structures, facilities and manpower) and policies (or lack thereof); and collectively they influence diabetes management. • The strategies and interventions that aim to improve diabetes management in Nepal need to carefully consider the deficiencies at the healthcare system level as well as at individual HCP level, both collectively as well as independently. • Improving outcomes for patients with chronic diseases such as T2D should be a healthcare system priority. Diabetes management in Nepal must shift from its current situation which focuses on episodic care to a comprehensive model of integrative medical care to achieve effective diabetes outcomes.
10	Gyawali et al. (29)	Current debate	To identify the problem and recommend solution for the growing burden of NCDS	• Healthcare is far from equitable and affordable; government spending on healthcare is very low and for NCDs specifically and is unevenly distributed across the country. • Healthcare is mainly financed by out-of-pocket expenditure, which is particularly difficult to bear for the poor people and this contributes to pronounced inequities in health. • Weak national health information and research system. • Interrupted supply of essential drugs for NCDs. • Lack of facilities and capacity for screening • Early diagnosis and effective management of NCDs within the PHC system. • Shortage and retention of health workforce, especially in rural areas and lack of retention. • The establishment of an NCD and Mental Health Section under the Department of Health Services is a positive development. However, efforts are needed for integration of NCD services in the mainstream care with strong focus on expanding community-level services for addressing NCDs.
11	Roglic et al. (49)	Editorial	To identify the burden, gaps, challenges, and ways forward.	• Early diagnosis and referral services are not available at all service levels, owing to lack of resources such as trained health professionals, NCD-related drugs and diagnostics. In turn, this leads to late diagnoses requiring tertiary-level care. • In response, Nepal has recently given heightened prominence through a national multisectoral action plan for prevention and control of NCDs. • With high-level political commitment, this plan aims to strengthen and orient health systems to address the prevention and control of NCDs and underlying social determinants, through people-centered primary health care and universal health coverage (UHC), strengthening capacity in primary care to diagnose and manage diabetes. • Greater political commitment is urged for investing more in health services and emphasize that national action plans for prevention and control of diabetes and other NCDs should be in line with strategies for health-system strengthening. Need a comprehensive framework to make our societies less diabetogenic, make our screening systems more patient-friendly, make our behavior-change interventions more powerful and sustainable, and make our health-care systems more effective to control diabetes and its complications.

The findings from the document review and IDIs and KIIs were triangulated. Four major themes emerged that were directly relevant to the implementation process of the existing policies and guidelines for diabetes management in the peripheral health system in Nepal. The key themes identified are: (i) limited implementation of policies into practice, (ii) lack of coordination among the different level of provider, (iii) lack of human resources for health and comprehensive quality services at the primary health care level, and (iv) boundaries for the people with diabetes to access diabetes care at primary health care and engagement to promote diabetes self-management.

### Implementation of Policies and Strategies Into Practice

In recent years, Nepal has developed policies for NCDs prevention and control. However, to date, no specific policies exist for the diabetes prevention and control. Some of the policies related to NCDs include (i) National Health Policy 2019, (ii) Multisectoral Action Plan for the Prevention and Control of Non-communicable Diseases (2014–2020), (iii) Nepal Health Sector Strategy (NHSS) Implementation Plan 2016–21, (iv) WHO's Package of Essential NCDs (PEN) Intervention at PHC setting, (v) Nepal Tobacco Products Control and Regulatory Act, 2011 (Smoke free zones/Tobacco Advertising), (vi) Food (Fifth Amendment) Rules, 2064 (2007) 2064.6.14 (1 Oct. 2007) (Packaging and labeling, (vii) Food Adulteration Act/), (viii) Alcohol Regulation and Control Policy, 2017, and (ix) The Public Health Service Act, 2018 (see Table 2).

The qualitative findings showed that most of the community health workers reported not using any protocol or guidelines in the management of diabetes. The understanding of package of essential NCDs (PEN) protocols was limited to few health care providers who received training on the PEN package in Nuwakot and Kavrepalanchowk districts. Most interviewed health care providers mentioned that they were unaware about any guidelines for diabetes management at the primary health care level, while few expressed that they had received basic training on PEN 2–3 years back in other districts. Some of the senior peripheral health care providers mentioned that there is a lack of guidelines for engaging community health workers for diabetes care in Nepal.

One of the participants said, “*We do not have such type of guidelines, protocols; however, we have heard from others or based on our own experiences, we have been providing some kind of diabetes relates services in particularly diabetes screening and distribution of basic medication” (ID: K003, CHW, Kavrepalanchowk)*.

Few health care providers mentioned that training for management of non-communicable disease including diabetes should focus on skill development rather than educating them on disease prevention and control and improving healthy behavior. Some participants expressed that the selection criteria for sending people for any training is not as per the guidelines for training and capacity building, rather selection of participant done solely based on nepotism and favoritism. This reflected the lack of implementation of National Health Training Center protocols. One health care provider reported- “*Two to three people have training on PEN package but there is no translation of learnings from training. I would say it's crucial to strengthen primary health system while providing training to the community health workers.” (NOO4, CHW, Nuwakot)*.

### Coordination and Collaborations Among the Different Level of Health Care Providers

Two studies suggested the need to improve the coordination among the different levels of the stakeholders (10, 25) with a greater involvement of relevant stakeholders from the government, civil society, and the private sector for early detection and management of diabetes in Nepal. This is also supported by our qualitative findings where majority of the participants reported the lack of coordination among the concerned stakeholders as a major problem for implementing any programs. Majority of the participants highlighted the need for an active engagement of all end-users from the inception to delivery and effective monitoring of the program. Some participants blamed the stakeholders for not being concerned for people's health. Additionally, few other stated political and vested interest of stakeholders as the impeding factor for coordination.

“*Engaging Management committee, health management committee, political representatives, teachers, health worker, FCHVs, etc. from the inception to delivery of the program will create ownership and trust toward the program activities. But unfortunately, INGOs/NGOs just collaborate with some powerful political leader or government representative and implement the program.” (ID:N003, CHW, Nuwakot)*

“*Though such programs are being carried out by many INGOs/NGOs, I think it should be carried out mobilizing employee by Nepal Government. If those programs can be conducted in collaboration with the Nepal government, INGOs, and NGOs it would be more fruitful and achievable.” (ID: N004, CHW Nuwakot)*

“*In community level we need to coordinate with FCHVs, our management committee which we have here, likewise health management committee, representatives, teachers, health workers etc. to make sustainable programs” (ID: K007, CHW, Kavrepalanchowk)*

Some health care providers from the Nuwakot district mentioned that there is a lack of monitoring and supervision of program related to NCDs. As such, one expressed- “*We have the PEN program, but nobody knows what we are doing. Focal person for NCDs does not have enough idea on NCDs. Honestly speaking, I think there isn't any monitoring done specifically for NCDs.” (N004, CHW, Nuwakot)*.

Few participants also cited lack of sensitization of issues related to NCDs, along with lack of technical and economic resources as major problems to deal with growing problem of diabetes and other NCDs.

### Primary Health Care Challenges in Delivering Comprehensive Care

All the included studies discussed the challenges that are prevalent with health care system in delivering diabetes care at primary health care level. Five out of eight studies mainly discussed the unavailability of the health resources and human resources at the primary health care system (9, 10, 25–27). Three studies reported sub-optimal readiness of public health care facilities in delivering services related to NCDs including diabetes (9, 25, 28). A literature review conducted by Gyawali et al. highlighted that public health facilities still highly prioritize communicable disease and maternal and child health services and are not well-prepared to deliver service for the people with NCDs including diabetes (10). Similarly, the study reported that the readiness of services to deliver comprehensive services in Nepal was sub-optimal because of lack of trained human resources, equipment, drugs, and standard guidelines for effective management of NCDs including diabetes (10). Likewise, an editorial paper highlighted several challenges such as, insufficient and/or inaccessible health care facilities offering diabetes services, inadequate diabetes trained manpower at primary health care and districts hospitals, inadequate team (medical office assigned for NCD, dietician, diabetes educator and nurses) at zonal and tertiary hospitals, and insufficient laboratories facilities to monitor beyond plasma sugar level in primary care in Nepal (27).

Moreover, WHO PEN program is the only nationwide robust program implemented in Nepal till date, however, it's implementation across the country is taking longer (26). Awareness, early diagnosis and prevention are the keys to diabetes management and control. Therefore, the government of Nepal has recognized a need to adopt a public health approach to halt the rise of diabetes and obesity (26). The management of diabetes in the peripheral health system is not comprehensive and are less focused on lifestyle modification, rather they are focused on providing the listed essential medicines (9).

Similarly, the findings of our study also noted the inadequate human resources for health and supplies of glucometers and strips required for screening of the diabetes cases. Likewise, higher proportion of the study participants reported the diabetes medicine, metformin, are unavailable in most of the health centers. However, few reported a limited stock of metformin in PHC level.

One health care provider said, “*Metformin was supplied by province government since last year, but not now and I don't know the reason” (ID: N004, CHW, Nuwakot)*.

Similarly, another health care provider expressed *- “We used to do glucometer testing previously but now we are out of strips.” (ID: N002, CHW, Nuwakot)*.

Majority of the health care providers expressed dissatisfaction with supply chain management system. Participants cited lack of co-ordination between health care coordinator and logistic division representative of district, inability of officials to forecast demand of medicines, inadequate funding and an inefficient purchasing system as the major reasons for inefficient supply chain management system. Most of the senior level health care professionals mentioned the lack of trained doctor or health care providers to manage Diabetes and other NCDs. One senior health care provider mentioned that- “*We envisioned the recruitment of human resources for health, such as doctor and health assistants but we cannot do anything because of lack of resources from federal and provincial government. District health office does not have budget to tackle NCDs specifically*.”* (DHO2, Nuwakot)*.

### Boundaries for the People With Diabetes to Access Diabetes Care at Primary Health Care

Three studies highlighted that people had low level of awareness on diabetes and its management (9, 10, 26). Studies included in this review showed that several challenges that impeded access to the diabetes care at the public health facility including PHC in Nepal, including the financial burden like out-of-pocket expenditures, inadequate essential drugs, lack of trust of people on government services, limited health care facilities and ineffective government-initiated health insurance system. Although primary health care is considered the backbone of health service delivery, this lacked adequate preventive and curative services for diabetes. Likewise the management of type 2 diabetes mellitus (T2DM) and its complications found to be a formidable challenge to the government owing to lack of appropriate infrastructure and financing scheme required to provide diabetes management services (10). Few health care providers also highlighted that there is lack of recognition of diabetes and major health related problems.

*One said “NCDs are a major health problem globally. Lack of recognition of these problems by them (ward / local government), not realizing that it's a major problem and design programs accordingly.” (ID: N004, CHW, Nuwakot)*

Similarly, barriers to engage in diabetes self-management practices included modifying the dietary habits that are deep rooted to cultural practices, unavailability of space for physical activity, lack of motivation toward lifestyle management, poor adherence to lifelong medication for diabetes and lack of family support in changing the behavior (9).

One participant expressed “*The major challenge is to change people's behavior. Most of the people are aware about the consequences and knowledgeable but they don't implement in their life. The main problem is the negligence or not prioritizing diabetes as a major health problem” (ID: K002, CHW, Kavrepalanchowk)*.

Some participants perceived a lower trust in the medicines supplied by the government because they believed that free medicines will not be effective on disease management. Few participants also perceived poor health literacy as a major challenge for diabetes prevention and management at the primary health care (PHC) level. One said:

“*Limited health literacy on diabetes among people is unaddressed issues. There is greater need of transforming the community through health literacy program. This could develop trust among people for government health facilities among those who do not feel so.” (ID:UM1, HC, Kavrepalanchowk)*

## Discussion

This study identified several key policies, strategies, and plans that have been formulated by the Government of Nepal and have aimed at addressing the problem of NCDs including diabetes in Nepal. The Government of Nepal has launched multisectoral Action Plan for the prevention and control of NCDs and implemented PEN package in selected districts of Nepal, there is still a lack of programs that emphasize screening, diagnosis of diabetes, and providing comprehensive self-management and care.

Given the increasing problem of NCDs including diabetes in Nepal, there has been a critical need for developing and implementing effective policies and strategies into affordable and feasible programs that can detect individuals at risk of developing diabetes, provide health services at the local level and can support people with diabetes in self-management of conditions. Examining available public health policies with regard to diabetes prevention and control is crucial, given the social and economic burden that diabetes creates for the individuals, communities and the health system as a whole. At present, Nepal has introduced a range of NCDs related policies, however they lack the specifics for diabetes prevention and control and the implementation of existing policies is weak.

Impact of some policies such as graphic warnings on tobacco packaging and alcohol products, diabetes risk factors survey, ban of alcohol advertisement and promotion and sponsorship are laudable but the implementation of policies for the prevention and management of type 2 diabetes at primary health care level is not yet well-organized. The poor implementation of policies for preventing and managing diabetes at the primary health care level have been largely reflected in recently published studies from Nepal (9, 29). The emerging evidence from Nepal during the COVID-19 pandemic have captured stories of people with diabetes who were deprived of basic primary health care services. This reflects poor implementation of different policies and programs to curb diabetes in Nepal. This argument is supported by a recent study which noted weak implementation of NCD related policies in LMICs compared to high-income countries (30). The success of the policies is determined based on the implementation process. There are many reasons for poor implementation of policies and strategies in low-income countries, including financial constraints, poor recognition of NCDs as the key priority health problem, inadequate trained human resources, and lack of guidelines and standard operating procedures, etc. (7). Therefore, the government should identify the strategies to facilitate an effective translation of the existing policies into practice. To start with, the government should engage external developmental partners, international non-governmental organizations, research and implementation organizations to put emphasis on strategies to curb growing burden of NCDs including diabetes (31). Moreover, political interest and support are essential to strengthening the implementation process of the existing NCD and diabetes related policies.

Studies have shown that delivery of diabetes care through strong PHC has resulted in lower health costs, better health outcomes, higher satisfaction of people with diabetes, fewer hospitalization rate, and greater socioeconomic equity (32–35). While the prevalence of diabetes and its impact on health and overall quality of life is high in Nepal, low institutional capacity of primary health care center, inadequate human resources for health, lack of resources/funding and priority for diabetes and other NCDs and poor intersectoral coordination are some key challenges to implementing programs for prevention and control of diabetes. Previous studies have noted lack of regular supply of medications, lack of glucometers required to screen diabetes, health education materials, and limited skills of health care providers for management of diabetes as major concerns at PHC level that impede delivery of diabetes care (7, 36, 37).

Lack of trained or poor retention of human resources for health is a pertinent issue in LMICs including Nepal, that needs to be addressed effectively in order to delivering diabetes prevention and care services in low-income settings (38, 39). These all can leave people with diabetes with no choice other than to seek medical services from a poorly regulated private sector which is generally means higher cost and is hence unaffordable for poor people (40, 41). Therefore, revitalization of primary health care is greatly required to deliver comprehensive diabetes prevention and management services along with appropriate support systems (in terms of guidelines, budget, trained human resources and logistics and supplies etc.) available at the primary care level. Also, it is crucial to establish referral services for the complicated diabetes cases from the PHC to secondary and tertiary level hospitals. Similarly, a comprehensive action plan to tackle diabetes with clear responsibilities and a monitoring plan backed by the necessary funds to support diabetes prevention and management programs at the community level is required. Further, monitoring and evaluation are vital for the quality of the care provided at the primary health care level along with strong national health information and research system in place in order to keep track of the ongoing services and patient follow up.

Previous studies have identified that importance of health literacy in improving behavioral, clinical, patient–provider communication, and other outcomes among people with diabetes (42, 43). It is obvious that people with adequate health literacy seek available health services, can effectively communicate with health care providers and manage their lifestyle required for maintaining good health. Evidence also suggests that health literacy is an important determinant of inequities in health (44). In most cases, poor health literacy is viewed as patient problem that can hinder obtaining information or communicating with health care providers. This would need further research in the case of Nepal. Therefore, diabetes prevention and control programs require comprehensive intervention strategies to tackle various issues related to prevention and care. This strategy would also help to reduce health disparities and establish people center care at primary health care center (44). Addressing the problem of diabetes across people of different strata, particularly among those with poor economic status, would need revisiting the existing health insurance policy of Nepal and improving the implementation process. The current health insurance policy in Nepal aims to address social inequity and breaking barriers to quality of care. The overall findings of the study suggest the need for developing context-specific cost-effective intervention approaches to prevention and control of NCDs including diabetes. Further, co-designing approach (45) could be adopted that will address the concerns of all stakeholders and design the people centered interventions that could be scalable to primary care level across the country.

### Research Implications

The findings of this study are useful for the policy makers in Nepal to map the gaps in formulation and implementation of NCDs related policies in Nepal, particularly diabetes prevention and control at the peripheral level. The findings are also helpful to strengthen the ongoing implementation of PEN package at the peripheral level. The lack of screening and awareness at the community level implies the greater need of people centered health literacy intervention (46) required to improve the health outcomes of the people living with diabetes through comprehensive self-management program. Similarly, there is a need for continuity of care for the people with diabetes which could include primary, secondary, and tertiary preventive approaches to maintain their blood sugar level and reduce the potential complications due to uncontrolled diabetes.

The primary health care has a significant role to play for the prevention and management of NCDs (47) including diabetes at the community level; however, resources and support provided to these care services now are inadequate. Given the importance of providing NCDs and diabetes related programs at the primary care level, there is a need for revitalizing the primary health care systems that effectively integrates the NCDs and diabetes related programs within the current health care systems.

#### Study Strengths and Limitations

As like other studies, this study does have strengths and some limitation. The strengths include: (i) study triangulated the findings from both literature review and a qualitative study conducted with health care providers and policy makers, (ii) findings gathered add to the literature on policy translation research on diabetes, and (iii) the gaps identified in the current study might guide the decision makers to take appropriate steps for diabetes prevention and management at primary health care setting of Nepal. Our study had some key limitations. First, search of literature was done in selected databases which may not have captured all the evidence. Second, some studies may have been missed to be included because of language bias as we did not include articles or policies available in Nepali language. Thirdly, the findings from a qualitative study cannot be generalized to other study settings of Nepal as they each constitute a small number of respondents.

## Conclusion

Our findings suggests that despite the formulation of a range of policies and strategies for the prevention and control of NCDs including diabetes in Nepal, there has been a poor translation of these policies into practices. Integrating NCDs including diabetes related programs within the current health care systems and revitalizing primary health care systems along with effective implementation of such programs are essential to achieving SDG-3, health, and wellbeing and ensuring universal access to health services by 2030. There is also a need for developing and implementing effective, context specific, and cost-effective intervention required for prevention and control of NCDs including diabetes in Nepal. In order to improve and strengthen implementation aspect of policies and strategies to NCDs and diabetes prevention and control in Nepal, there is a need for engaging wider stakeholders such as government, health care professionals, local community leaders, political leaders, private sectors etc.

## Data Availability Statement

The raw data supporting the conclusions of this article will be made available by the authors, without undue reservation.

## Ethics Statement

The studies involving human participants were reviewed and approved by Human research Ethics Committee of Nepal Health Research Council Nepal. The patients/participants provided their written informed consent to participate in this study.

## Author Contributions

RS, UY, AbS, GP, and LR contributed conceptualizing the study, drafting the manuscript, and finalization. RS, AbS, DM, GP, BK, HI, UY, LR, and RK contributed to data collection, processing, analyses, and results' write up. UY, PP, YH, AbS, ArS, YH, BK, RK, TS, and LR thoroughly reviewed the manuscript and contributed substantially for necessary revision. RS, UY, AbS, GP, and LR final reviewed the manuscript and prepared for submission. All authors contributed to the article and approved the submitted version.

## Funding

RS, DM, and HI are regular project staff of the project funded by Japan Agency for Medical Research and Development (AMED). LR, TS, BK, AbS, ArS and RK are research investigators of the ongoing project funded by AMED Japan (Grant Number JP21jk0110020) under the Global Alliance for Chronic Disease (GACD) Call and funding mechanism.

## Conflict of Interest

The authors declare that the research was conducted in the absence of any commercial or financial relationships that could be construed as a potential conflict of interest.

## Publisher's Note

All claims expressed in this article are solely those of the authors and do not necessarily represent those of their affiliated organizations, or those of the publisher, the editors and the reviewers. Any product that may be evaluated in this article, or claim that may be made by its manufacturer, is not guaranteed or endorsed by the publisher.
